# Supplementary material for: Tat-Beclin-1 Peptide Ameliorates Metabolic Dysfunction-Associated Steatotic Liver Disease by Enhancing Hepatic Autophagy
Source: Int J Mol Sci. 2024 Nov 18;25(22):12372. doi: 10.3390/ijms252212372 (PMC11594940; doi:10.3390/ijms252212372)
Supplement: Supplementary file 1 [file ijms-25-12372-s001.zip › ijms-3268911-supplementary.pdf]

Supplementary Data

Table S1. Quantitative RT- PCR primer sequences for fatty acid oxidation-related genes and pro-inflammatory cytokine genes in mice.

Gene		Sequence 5'---3'
Gapdh	Forward	CATGTTCCAGTATGACTCCACTC
	Reverse	GGCCTCACCCCATTTGATGT
**Fatty acid oxidation**		
Ucp2	Forward	GCTGGTGGTGGTCGGAGATA
	Reverse	ACTGGCCCAAGGCAGAGTT
Lcad	Forward	GGAGTAAGAACGAACGCCAA
	Reverse	GCCACGACGATCACGAGAT
Cpt-1α	Forward	AGGACCCTGAGGCATCTATT
	Reverse	ATGACCTCCTGGCATTCTCC
Acox	Forward	CGGAAGATACATAAAGGAGACC
	Reverse	AAGTAGGACACCATACCACCC
Ppar-α	Forward	TATTCGGCTGAAGCTGGTGTAC
	Reverse	CTGGCATTTGTTCCGGTTCT
**Pro-inflammatory cytokines**		
Il-1β	Forward	CCGTGGACCTTCCAGGATGA

	Reverse	GGGAACGTCACACACCAGCA
Tnf-α	Forward	CATCTTCTCAAAATTCGAGTGACAA
	Reverse	TGGGAGTAGACAAGGTACAACCC

Abbreviations: *Gapdh*, glyceraldehyde 3-phosphate dehydrogenase; *Ucp2*, uncoupling protein 2; *Lcad*, long chain acyl-CoA dehydrogenase; *Cpt-1α,* carnitine palmitoyl-transferase 1α; *Acox*, acyl-CoA oxidase; *Ppar-α*, peroxisome proliferator-activated receptor α; *IL-1β*, interleukin 1β; *Tnf-α*, tumor necrosis factor α.

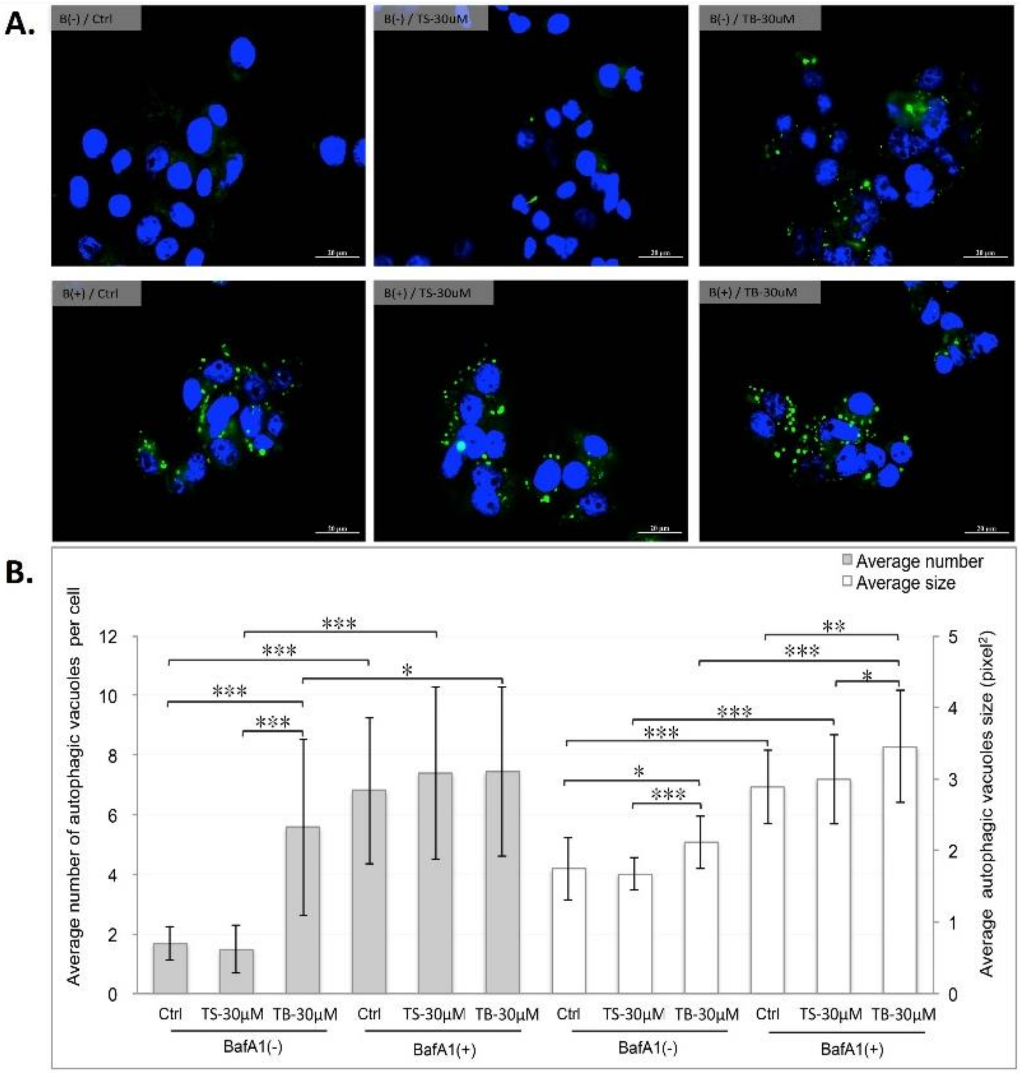

Figure S1. TB-1 peptide induces autophagic vacuoles formation in HepG2 cells.

HepG2 cells were treated with 30 µM of either the TS or TB-1 peptide for 4 hours, followed by the addition of 400 nM bafilomycin A1 or no addition 2 hours before staining autophagic vacuoles using the CYTO-ID® Autophagy Detection Kit (A). The average number and size of autophagic vacuoles (green fluorescence) were quantified using ImageJ software (version 1.53k) (B). Data are presented as mean ± SD ($n = 3$ per group). Statistical significance: $^{*}p < 0.05$, $^{**}p < 0.01$ and $^{***}p < 0.001$.

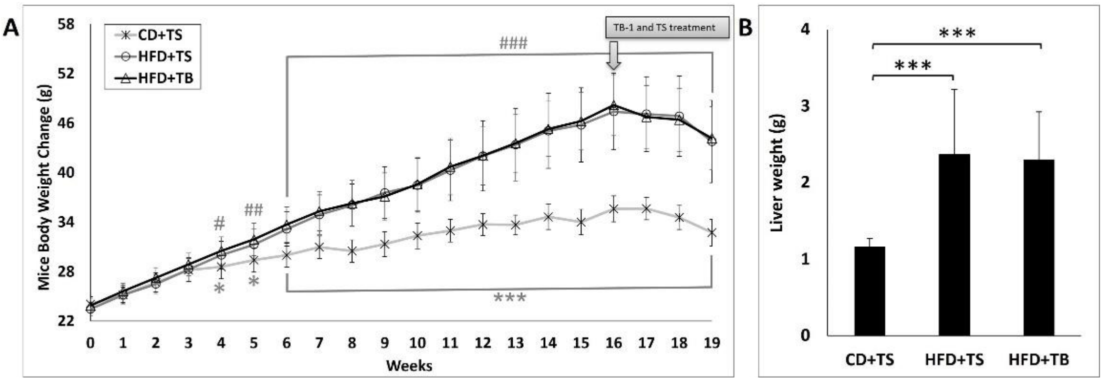

Figure S2. TB-1 peptide shows no effects on body and liver weight in the HFD-induced murine MASLD model. Mice were treated with either TB-1 or TS peptide three times per week for three consecutive weeks (a total of nine treatments). Body weight was recorded weekly (A), and liver weight was measured post-sacrifice (B). Sample size: $n = 10$ per group Statistical significance: $^{*}p < 0.05$ and $^{***}p < 0.001$ (HFD + TS control versus CD + TS control) and $^{\#}p < 0.05$, $^{\#\#}p < 0.01$ and $^{\#\#\#}p < 0.001$ (HFD + TB-1 versus CD + TS control). Abbreviations: CD: chow diet; HFD: high fat diet; TS: Tat scrambled control peptide; TB: Tat-Beclin-1 peptide.

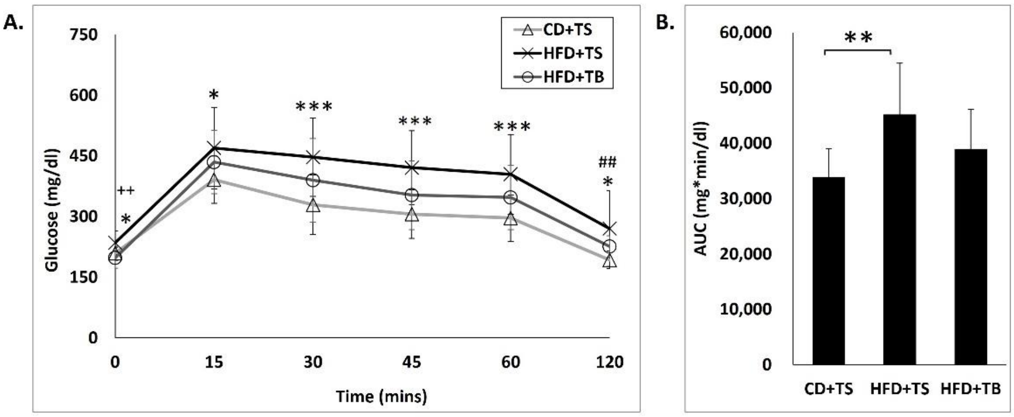

Figure S3. TB-1 peptide exhibits mild effects on glucose profiles of OGTT in the HFD-induced murine MASLD model. (A) The OGTT curve illustrates changes in blood glucose levels at various time points. (B) The bar graph represents the area under the curve (AUC) for the OGTT. Sample size: $n = 10$ per group. Statistical significance: $^{*}p$ <0.05, $^{**}p$ < 0.01 and $^{***}p$ < 0.001 (HFD + TS control versus CD + TS control)；$^{\#\#}p$ <0.01 (HFD + TB-1 versus CD + TS control), and ^{++}p <0.01 (HFD + TB-1 versus HFD + TS control). Abbreviations: CD: chow diet; HFD: high-fat diet; OGTT: oral glucose tolerance test; AUC: area under the curve; TS: Tat scrambled control peptide; TB: Tat-Beclin-1 peptide.

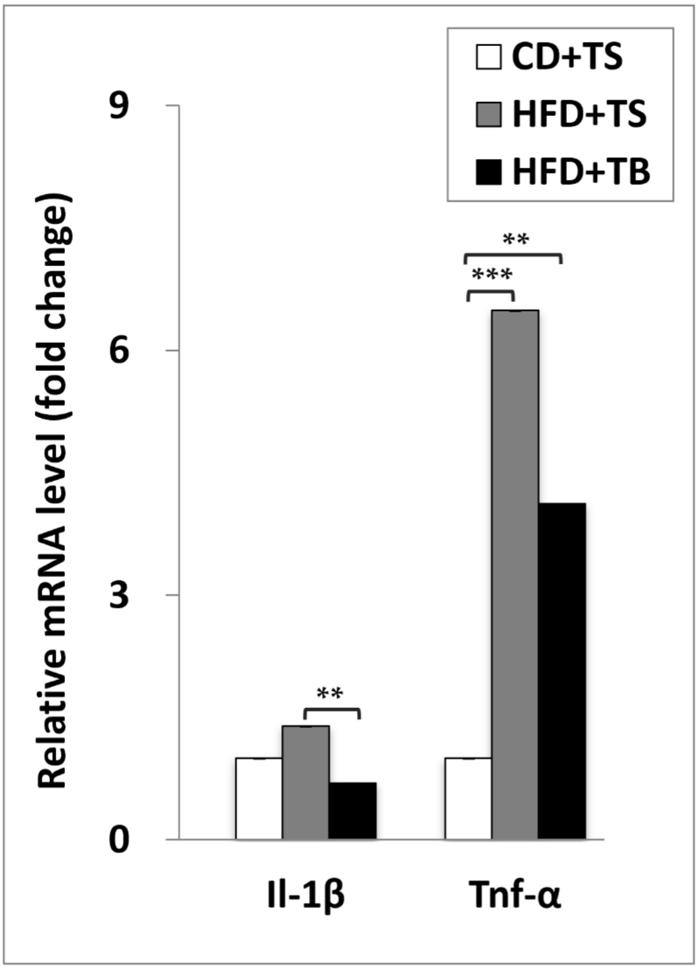

Figure S4. Effects of TB-1 peptide on liver inflammation. Expression levels of Pro-inflammatory cytokines genes in the liver of the HFD-induced murine MASLD model. All data are presented as fold changes compared to the expression level of the CD + TS group (n = 10 per group). Statistical significance: ** $p < 0.01$ and *** $p < 0.001$. Abbreviations: CD, chow diet; HFD, high-fat diet; TS, Tat scrambled control peptide; TB, Tat-Beclin-1 peptide. *IL-1β,* interleukin 1β; *Tnf-α,* tumor necrosis factor α.
